# Response to Letter to the Editor regarding: “Log transformation of proficiency testing data on the content of genetically modified organisms in food and feed samples: is it justified?”

**DOI:** 10.1007/s00216-020-02639-z

**Published:** 2020-04-21

**Authors:** Wim Broothaerts, Fernando Cordeiro, Philippe Corbisier, Piotr Robouch, Hendrik Emons

**Affiliations:** European Commission, Joint Research Centre (JRC), Geel, Belgium

Dear Editor,

The European Union Reference Laboratory for Genetically Modified Food and Feed (EURL GMFF) is organizing regularly, in line with its mandate under Regulation (EU) 2017/625, Proficiency Testing rounds (PTs) primarily for EU National Reference Laboratories (NRLs) appointed in the frame of official controls for the determination of GMOs in different food and feed commodities. For more than a decade, this EURL/NRLs network is implementing fit-for-purpose PCR methods, validated by the EURL GMFF with participation of selected NRLs, is providing common analytical guidance and training, and is sharing harmonized analytical procedures with the whole European Network of GMO Laboratories (ENGL) [1]. Hence, Broothaerts et al. [2] have considered a population of competent and experienced laboratories capable of delivering reliable results for each PT round, regardless of the specifically addressed PCR target. Consequently, it had been expected that the collected results were normally distributed, which constituted the null hypothesis in the study.

In the Letter to the Editor, the authors justify the application of a log transformation to GMO PT data from the Fapas^®^ GM PT scheme with how a PCR method is generating the results, i.e., with the “sequential multiplication of small numbers of genomes with responses converted to quantities of GMO material via a log-linear calibration.” However, the numbers of genomic targets multiplied during PCR are generally in the order of hundred thousands for the taxon-specific target and over 100 for the GM target, which should not be considered “small numbers”. Furthermore, the PCR amplification step itself is only part of the analytical procedure, which starts with sub-sampling, DNA extraction, total DNA quantification, and testing for the absence of PCR inhibitors in the extracted DNA. Among others, the contribution from partial inhibition to the variation of results reported by participants can be significant, particularly when dealing with complex food or feed matrices. Overall, there are linear and non-linear concentration dependencies involved in a whole GMO quantification process so that the contribution from the exponential amplification of targets to the data distribution should not be overstressed.

The rational for suggesting the statistical design to be applied in the PTs organized by the EURL GMFF has been described extensively in the paper [2]. Furthermore, the authors would like to draw the attention of the Editor to the following issues:

- The outlier test was applied for each PT round, to all reported PT values. This approach is based on the commonly accepted assumption that “a set of reported PT values (results) from competent participants will be approximately normally distributed, or at least unimodal and reasonable symmetric” [3§5.3]. “The distribution of results from competently determined measurements is mixed (or ‘contaminated’) with ‘erroneous’ results which may be identified as outliers” [3 §5.3]. The chosen outlier test applied for all PTs identified “values deviating from the robust mean by more than 3 times the robust standard deviation” [3 §6.6.3 Note 3]. The Shapiro-Wilk test used to assess the departure from normality is extremely sensitive to any extreme value present in the distribution. The other statistical tests applied (Kolmogorov-Smirnov test or the combination of skewness and kurtosis tests) could not identify any significant departure from normality for any of the datasets studied [2].

Sykes and MacArthur refer to a “more appropriate” outlier test without providing further details.

b) The Shapiro-Wilk test for normality requires a “considerable computational effort” [4], and tables for the critical values are obtained in statistical textbooks starting from *n* = 3 [4]. This table of critical values is embedded in the software used (Statistica 13.5, Tibco Software). Our datasets include 30 to 60 values, thus well above the minimum tabulated value to draw reliable conclusions about normality. Any departure from normality was scrutinized taking into account the corresponding Shapiro-Wilk test (W) value and comparing it with the critical value (Wc, for a given population *n* and at 95% confidence level). Any departure from normality was considered significant if W ≥ Wc.
c) Based on the statistical tests performed, we concluded that the log-data transformation was not justified for most data distributions. All deviations from normality were systematically scrutinized. After rejection of the laboratory results derived from methods measuring an unreliable target (e.g., adh1–70 bp), the remaining datasets proved once again to be normally distributed.
d) Sykes and Macarthur simulated “log-normal” datasets to claim the failure of the statistical analysis applied by us. This is certainly not convincing, since the same “erroneous” conclusion could be obtained starting from a genuine normal distribution (when randomly selecting 50 data out of 1000). It should be stressed that the data in [2] are not simulated data but really reported PT results.

Other authors pointed already out that the “log-transformation of data distributions that appear skewed may not be justified” [5, 6]. Our paper shows that the reported GMO results generated by quantitative PCR methods are normally distributed. This could have been facilitated by the experience of the well-trained laboratories participating to our PTs. PT results reported by other laboratories (with a broad variety of expertise) may deviate from such a scenario. However, this does not mean that the results have to be (log) transformed. A deviation from normality may indicate the need to investigate experimental issues of the specific analytical procedures used by the PT participants.
